# Discrepancies in indel software resolution with somatic CRISPR/Cas9 tumorigenesis models

**DOI:** 10.1038/s41598-023-41109-1

**Published:** 2023-09-08

**Authors:** Qierra R. Brockman, Amanda Scherer, Gavin R. McGivney, Wade R. Gutierrez, Jeffrey Rytlewski, Alexa Sheehan, Akshaya Warrier, Emily A. Laverty, Grace Roughton, Nina C. Carnevale, Vickie Knepper-Adrian, Rebecca D. Dodd

**Affiliations:** 1 Department of Internal Medicine, Carver College of Medicine, University of Iowa, 375 Newton Rd, 5206 MERF, Iowa City, IA 52246 USA; 2 Holden Comprehensive Cancer Center, University of Iowa, Iowa City, IA USA; 3 Cancer Biology Training Program, University of Iowa, Iowa City, IA USA; 4 Department of Molecular Physiology and Biophysics, University of Iowa, Iowa City, IA USA; 5 Medical Scientist Training Program, University of Iowa, Iowa City, IA USA

**Keywords:** Software, Experimental organisms

## Abstract

CRISPR/Cas9 gene editing has evolved from a simple laboratory tool to a powerful method of in vivo genomic engineering. As the applications of CRISPR/Cas9 technology have grown, the need to characterize the breadth and depth of indels generated by editing has expanded. Traditionally, investigators use one of several publicly-available platforms to determine CRISPR/Cas9-induced indels in an edited sample. However, to our knowledge, there has not been a cross-platform comparison of available indel analysis software in samples generated from somatic in vivo mouse models. Our group has pioneered using CRISPR/Cas9 to generate somatic primary mouse models of malignant peripheral nerve sheath tumors (MPNSTs) through genetic editing of *Nf1*. Here, we used sequencing data from the in vivo editing of the *Nf1* gene in our CRISPR/Cas9 tumorigenesis model to directly compare results across four different software platforms. By analyzing the same genetic target across a wide panel of cell lines with the same sequence file, we are able to draw systematic conclusions about the differences in these software programs for analysis of in vivo-generated indels. Surprisingly, we report high variability in the reported number, size, and frequency of indels across each software platform. These data highlight the importance of selecting indel analysis platforms specific to the context that the gene editing approach is being applied. Taken together, this analysis shows that different software platforms can report widely divergent indel data from the same sample, particularly if larger indels are present, which are common in somatic, in vivo CRISPR/Cas9 tumor models.

## Introduction

Clustered regularly interspaced short palindromic repeat (CRISPR) sequences were first studied nearly 30 years ago^1^. Further characterization of CRISPR sequences and Cas genes demonstrated the adaptive immune, anti-viral function of Cas9 nuclease activity that was later harnessed as a powerful genome editing tool^2–4^. In 2013, Zhang and colleagues adapted the CRISPR/Cas9 system for genome editing of eukaryotic cells^5^. This work was essential to unlocking the genomic editing power of the CRISPR/Cas9 system as we know it today. Currently, CRISPR/Cas9 technology has evolved from a simple tool used to facilitate laboratory studies to a powerful instrument driving novel clinical therapeutics. Notably, CRISPR/Cas9 technology has shown clinical utility in a number of cancer types with additional clinical trials ongoing (www.clinicaltrials.gov).

One bottleneck of CRISPR/Cas9 technology is the ability to accurately characterize the indels and/or specific mutations generated by gene editing^6,7^. Following the commercialization of CRISPR/Cas9, there was a drastic increase in the number of publicly available platforms to assist with optimization of gRNA design and indel analysis. The gold standard for CRISPR indel analysis in the clinic is next generation sequencing (NGS). However, for labs that use CRISPR at high volumes to model patient disease and screen new therapeutic options, NGS is not a cost or time effective approach. The two most common indel analysis platforms are TIDE (Tracking of Indels by Decomposition) and Synthego. The utility of these platforms has been extensively reported for in vitro use and the generation of transgenic mouse models^8^. Comparative analysis of TIDE and Synthego in cultured cells demonstrated that both algorithms strongly correlate with NGS^9^. However, the growing applications and increased clinical trial presence of CRISPR/Cas9 technology highlight the need to better understand CRISPR/Cas9 efficiency in contexts beyond cells in a dish.

Our group was one of the first to use CRISPR/Cas9 to generate somatic primary mouse models of soft tissue sarcomas, including malignant peripheral nerve sheath tumors (MPNSTs)^10–12^. MPNSTs are an aggressive subtype of soft tissue sarcoma that arise from the myelinating nerve sheath of peripheral neurons following loss of key tumor suppressor genes including neurofibromin 1 (*NF1*) and *p53*. Loss of *Nf1* is a hallmark of MPNST biology, and is required for MPNST development in our model and other transgenic MPNST mouse models^13–19^. In this CRISPR/Cas9 tumorigenesis model, adenovirus containing Cas9 and guide RNAs (gRNA) directed at *Nf1* and *p53* is directly injected into the sciatic nerve^10–12,20^. De novo tumors with clinically relevant mutations develop 3–4 months later and are used to study MPNST progression and identify novel, targeted therapies. Other groups have used similar CRISPR/Cas9-based approaches to generate novel somatic models of lung, liver, pancreatic and other cancer types^21–28^. Importantly, all of these models use CRISPR/Cas9 to induce somatic mutations in vivo, which are more complex than indels generated in vitro.

One of the first steps in characterizing these in vivo tumor models is to define the indel patterns generated by CRISPR/Cas9 editing in tumor-derived tissue via sanger sequencing. In the past few years, there have been multiple in silico software launched to aide researchers in various steps of the CRISPR gene editing workflow^6,29^. There are several publicly-available programs designed to deconvolute sanger sequencing files to predict CRISPR/Cas9-induced indel types and percentages in an edited sample. However, to our knowledge, there has not been a cross-platform comparison of available indel analysis software in samples generated from somatic in vivo mouse models.

In this study, we directly compare four widely-used indel software packages including TIDE^30^, Synthego^31^, DECODR^32^, and Indigo^33–35^. Each of these software packages have different input methods, readouts, and algorithms used to report indel properties. Common outputs include number of indels, indel size, and percentage of indel composition found within an individual sample. We used sequencing data from in vivo editing of the *Nf1* gene in our CRISPR/Cas9 tumorigenesis model to analyze indels detected across the different software platforms. By analyzing the same genetic target across a wide panel of samples with the same sequence file, we are able to draw systematic conclusions about the differences of these software programs for analysis of in vivo-generated indels. We identified strong variability in data reported from different software packages, including discrepancies in the number, size, and frequency of indels across *Nf1* sequencing data from MPNSTs generated in four classically inbred strains. These data highlight the importance of selecting indel analysis platforms specific to the context that the gene editing approach is being applied.

## Materials and methods

### Samples

MPNSTs were made using our previously published CRISPR/Cas9 tumorigenesis model with gRNAs directed at neurofibromin 1 (*Nf1*) and tumor suppressor p53 (*Trp53*)^20^. Adenovirus (Ad) containing Cas9 and gRNAs targeting *Nf1* and *p53* were purchased from ViraQuest. Prior to injection, Ad CRISPR constructs were mixed with DMEM and calcium phosphate. Next, 25 µL of prepared virus was injected into directly into the sciatic nerve (SN) of mice. Tumor volumes were monitored 3 times weekly until reaching a predetermined terminal volume of 1500 mm^3^, in accordance with IACUC guidelines at the University of Iowa. Tumors were harvested when they reached 1500 mm^3^ and primary tumor tissue was collected for molecular analysis, histology, and generation of cell lines.

Cell lines were derived from terminally-harvested MPNSTs. Tumors were finely minced and digested in dissociation buffer Collagenase Type IV (700 units/mL, Thermo, 17104-019, Thermo Fisher Scientific, Waltham, MA, USA) and dispase (2.4 units/mL, Thermo, 17105-041, Thermo Fisher Scientific, Waltham, MA, USA) in PBS for 1–1.5 h at 37 °C on an orbital shaker as previously published^20^. Dissociated tissue was passed through a sterile 70 µM cell strainer (Fisherbrand, 22363548, Thermo Fisher Scientific, Waltham, MA, USA), washed once with PBS, and resuspended in DMEM (Gibco, 11965-092, Thermo Fisher Scientific, Waltham, MA, USA). Cells were cultured in DMEM containing 10% FBS, 1% penicillin–streptomycin (Gibco, 15140-122, Thermo Fisher Scientific, Waltham, MA, USA) and 1% sodium pyruvate (Gibco, 11360-070, Thermo Fisher Scientific, Waltham, MA, USA). After 10 passages, cells were used for indel analysis and subsequent studies.

**Figure 1:**
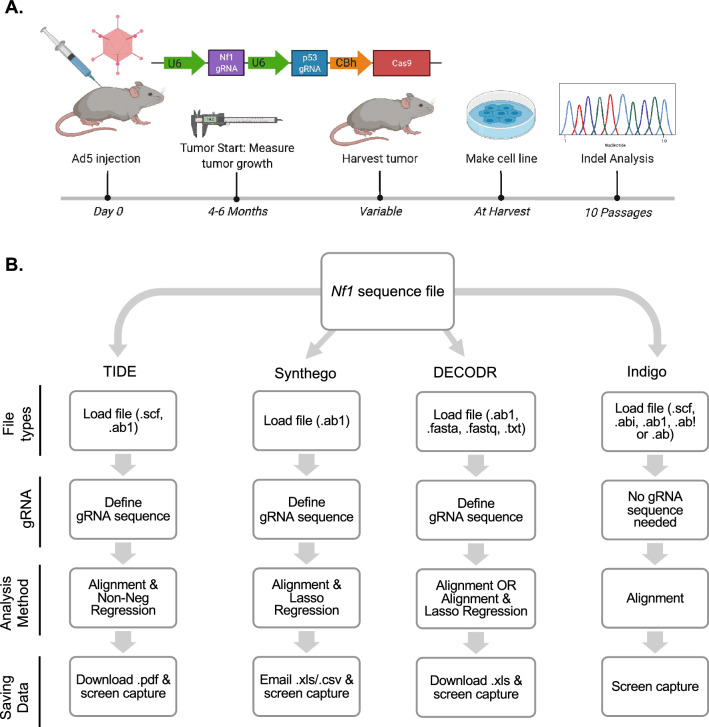
Model of CRISPR/Cas9 tumorigenesis model and sequence collection and processing. (**A**) Workflow of CRISPR/Cas9 de novo MPNST development, cell line generation, and sequence analysis. (**B**) Schematic of key features in the four platforms used for sequence/indel analysis.

Genomic DNA sequences were obtained from previously-published cell lines and sanger sequences from cell lines derived from primary tumors (Fig. 1)^20^. Nf1 and p53 genomic sequences that span the gRNA targeted region were amplified by PCR using Phusion high-fidelity DNA polymerase (NEB, M0530L). Primer sequences can be found in our previous publication^20^. PCR amplicons were purified with the Monarch PCR and DNA Cleanup Kit (NEB T1030S). Sanger sequencing was performed by the Genomics Division of the Iowa Institute of Human Genetics at the University of Iowa. Indel frequencies were quantified from the chromatograms by sequence trace analysis using TIDE, Synthego, DECODR, and Indigo (for select sequences).

### Indel analysis

*Nf1* sequencing files were input into various indel analysis software, along with gRNA sequence and wild-type control sequencing files, to determine indel detection differences between software in a primary tumorigenesis model. Samples were compared to a wild-type control sequence of *Nf1* of their respective background strain (Fig. 1; Supplementary Fig. 1; Table 1). Indel analysis software included TIDE, Synthego, DECODR, and Indigo (for select sequences). All software included alignment functions while having varying algorithms to predict indel percentage. Select indel analysis was conducted on *p53* sequences from corresponding biological samples.

**Table 1:** Indel analysis software feature comparison.

Feature	TIDE	Synthego	DECODR	INDIGO
Alignment	+	+	+	+
Indel contribution	+	+	+	−
Algorithm	Non-Neg regression	Lasso regression	Lasso regression	−
Alignment Window	Adjustable	Not adjustable	Not adjustable	Not adjustable
Default Inference Window (bp)	− 50 to + 15	− 30 to + 14	No limit	No limit
# Of gRNAs	1	1	2	−
Suggested Application	In vitro editing only	In vitro editing only	Somatic in vivo or in vitro editing	Not recommended

#### TIDE

Tracking of Indels by Decomposition (TIDE) is a publicly available web-based software designed to identify and quantify indels. In the user interface of TIDE, you upload either a .scf or .ab1 file type and the gRNA sequence of interest. The sequences are aligned and then the data are analyzed using a non-negative regression. The data can be saved as a .pdf for raw information while some sequence features can only be saved via screen capture (Fig. 1; Supplementary Fig. 1; Table 1).

#### ICE Synthego

Synthego is a publicly available web-based software designed to identify and quantify indels. In the user interface of Synthego, you upload .ab1 file types and the gRNA sequence of interest. The sequences are aligned and then the data are analyzed using a lasso regression. The data can be saved as a .xls or .csv for raw information while some sequence features can only be saved via screen capture (Fig. 1; Supplementary Fig. 1; Table 1).

#### DECODR

DECODR is a publicly available web-based software designed to identify and quantify indels. In the user interface of DECODR, you upload either a .fasta, .fastq, .ab1, or .txt file type and 1–2 gRNA sequence of interest. The sequences are aligned and then the data are analyzed using a lasso regression. The data can be saved as a .xls for raw information while some sequence features can only be saved via screen capture (Fig. 1; Supplementary Fig. 1; Table 1).

#### Indigo

Indigo is a publicly available web-based software alignment tool that can identify indels and predict homozygosity or heterozygosity. In the user interface of Indigo, you upload either a .scf, .abi, .ab1, .ab!, or .ab file type. The gRNA sequence of interest is not defined. The sequences are aligned and then the data are reported as the entire sequence of every different variant detected in the uploaded sequence. The data can be saved by copying sequences into a word or .txt file while some sequence features can only be saved via screen capture (Fig. 1; Supplementary Fig. 1).

### Next generation sequencing (NGS)

PCR-amplified *Nf1* products from the seven variable sequences were randomly sheared into 350 bp fragments through ultrasonic disruptors, then end repaired, A-tailed, and further ligated with Illumina adapters. The fragments with adapters were size-selected, PCR amplified, and purified. The library was checked with Qubit and real-time PCR for quantification and bioanalyzer for size distribution detection. Quantified libraries will be pooled and sequenced on Illumina NovaSeq6000 PE150 platforms, according to effective library concentration and data amount required. Fastq files sequence quality was confirmed via FastQC. Sequences with a per base quality score over 28 were retained for downstream analysis. Phred quality scores were checked for error probability in base calling (≥ 30). Next, sequences were aligned and indexed via Galaxy workflow, BWA-MEM2. Bam and bai files were input into IGV_2.16.1 to visualize alignments. Finally, absolute max indel sizes observed via NGS were compared to the max indel sizes observed via TIDE, Synthego, and DECODR for each sample.

### Statistical analysis

Statistical analysis was performed using the Prism 9 software (GraphPad), and a p-value < 0.05 was considered statistically significant. Analysis of total indel percentage was analyzed with a paired-ANOVA with Tukey’s multiple comparisons.

### Study approval

All animal procedures for this study were approved by the Institutional Animal Care and Use Committee (IACUC) at University of Iowa, Iowa City, Iowa, USA and were carried out in accordance with ARRIVE guidelines. All methods were carried out in accordance with AVMA guidelines, and are consistent with the commonly accepted norms of veterinary best practice.

## Results

### Indel detection varies across indel analysis software

To assess the outputs between indel analysis software, we generated cell lines from 18 primary CRISPR/Cas9-generated tumors (Fig. 1, Supplementary Fig. 1). Importantly, all of these tumors were generated from identical guide RNAs targeting *Nf1* and *Trp53*. Following ten passages, we extracted genomic DNA from the cell lines and PCR amplified the *Nf1* gene for sanger sequencing^20^. We next used the same sanger sequencing file of *Nf1* for systematic analysis by TIDE, Synthego, DECODR, and Indigo alignment indel analysis software. We analyzed the first 10 samples with all four programs. However, results from Indigo were difficult to summarize and compare to the other programs, as Indigo does not have sequence deconvolution capabilities. Therefore, the remaining 8 sequences were analyzed using only TIDE, Synthego, and DECODR.

**Figure 2:**
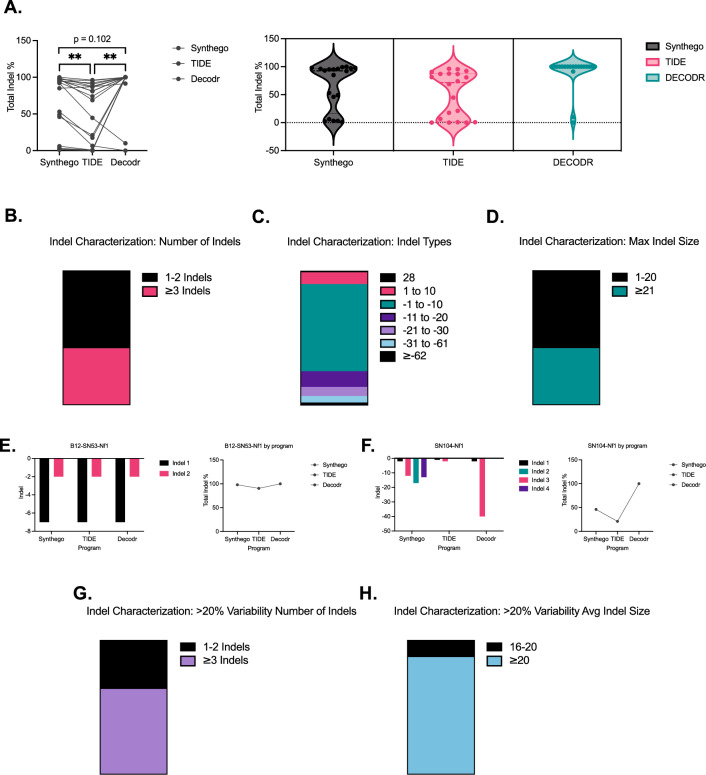
Mutational landscape correlates with the variability of indel analysis software. (**A**) Total indel percentage of *Nf1* sequences on Synthego, TIDE, and DECODR (n = 18). Variability in total indel percentage was analyzed by one-way ANOVA with Tukey’s multiple comparisons. (**B**,**D**) Global indel characterization of number of indels (**B**), indel type (**C**), and maximum indel size detected (**D**). (**E**) Representative indel characterization of a *Nf1* sequence with a simple mutational landscape of 1–2 indels detected. (**F**) Representative indel characterization of a *Nf1* sequence with a complex mutational landscape of ≥ 3 indels detected. (**G**) Global number of indel characterization of sequences that had greater than 20% variability across platforms. (**H**) Global indel size characterization of sequences that had greater than 20% variability across platforms. Data represents biological replicates with the mean ± SD; *P < 0.05, **P < 0.01, ***P < 0.001, ****P < 0.0001.

We first evaluated the traditional outputs from indel analysis software programs including the total number of indels, the size of each detected indels, and the percent composition of each indel detected within the sample. *Nf1* sequences from CRISPR/Cas9-derived tumors were input into either Synthego, TIDE, or DECODR to characterize indels. Surprisingly, different analyses reported widely divergent amounts of total indel percentage within the same sample, with significant differences detected between TIDE and the other programs (Fig. 2A, left). Furthermore, the distribution of total indel percentage in each sample was different depending on the software package (Fig. 2A, right). For Synthego, indels were reported with a trimodal distribution, while TIDE identified indels in a bimodal distribution. In contrast, DECODR analysis was heavily skewed towards reporting 100% indel composition.

Upon further characterization, we noticed that the type of indels identified fell into two distinct mutational profiles. Approximately two-thirds (58%) of the sequences identified only had 1–2 indels, while the remaining one-third (42%) of the sequences had 3 or more indels identified, with the most complex mutational landscape having as many as 6 different indels (Fig. 2B, Supplementary Fig. 3). While the majority of indels involved 1–10 base pair deletions, we identified a wide spectrum of indels ranging from 28-base pair insertions to deletions of 62 or more base pairs (Fig. 2C). Similar to patterns observed for the number of indels, the maximum absolute indel sizes detected fell into two categories with ~ 60% of sequences having indels of 1–20 base pairs and the remaining ~ 40% of sequences having a maximum indel size > 20 base pairs, with indels ranging up to 150 base pairs (Fig. 2D).

When looking at individual samples, we observed less variability in total indel percentage across indel analysis software for sequences that contained fewer indels of smaller sizes. This scenario is illustrated in Fig. 2E where all three software packages detected the same two indels of − 2 and − 7. Conversely, sequences with more and/or larger indels were more variable in total indel percentage. In the example shown in Fig. 2F, Synthego identifies 4 indels of moderate size, TIDE identifies 2 small indels, and DECODR identified 2 indels, including a 40 base pair deletion. The corresponding total indel percentage varies wildly across platforms for this sample, reflecting the different indel patterns identified from each software package. Similar detailed analysis of the remaining 16 samples in this study can be found in Supplementary Data for samples with 1 to 2 indels (n = 10 samples, Supplementary Fig. 2) and samples with ≥ 3 indels (n = 6 samples, Supplementary Fig. 3). After analyzing Nf1 indels in all 18 cell lines across the 3 analysis programs, we determined that the majority of samples with > 20% variability across different software had ≥ 3 indels with the average indel size > 20 base pairs (Fig. 2G,H).

### Indel analysis software variability of total indel percentage correlates with indel size

**Figure 3:**
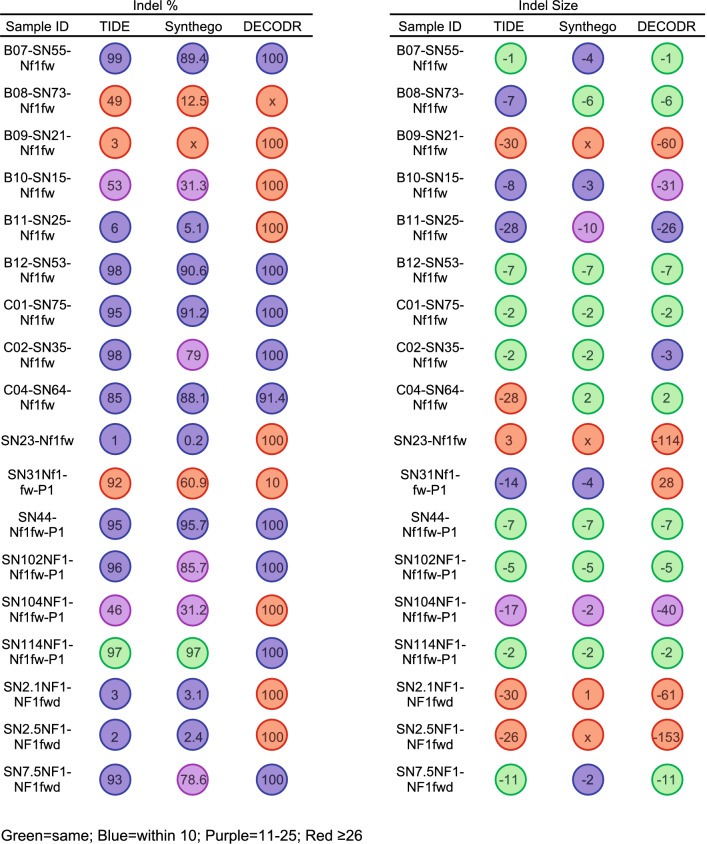
Indel analysis software variability of total indel percentage correlates with indel size. Total indel percentage and indel size for each sequence for all three platforms. Total indel percentages and indel sizes are color coded with different circle colors: green circles indicate the value was the same across platforms, blue circles indicate the value was within 10 across platforms, purple circles indicate the value was between 11 and 25 from other platforms, and red circles indicate a variation greater than 26.

To visualize the differences between indel analysis software across all 3 analyses for the same sample, we reported the indel percentage and indel size for each program side-by-side (Fig. 3). Values that matched the other two programs are color-coded with a green bubble while values that were within either 10% or 10 base pairs (bp) are color-coded with a blue bubble. Similarly, values within 11–25% or 11–25 bp are represented with a purple bubble, and values with a difference > 26% or > 26 bp are represented with a red bubble.

For the majority of *Nf1* samples, we observed that indel percentage and indel size positively correlated. In general, samples with good correlation of indel percentage also had strong concordance in indel size, although this was not always the case (i.e.: sample B08-SN73). We observed higher concordance across the different software packages when indels were smaller. Samples with green or blue indel size across all 3 platforms were between − 11 and + 2. Similarly, samples with green or blue percent indel cuts were all > 85%, and all but one showed strong concordance also among indel size, ranging from − 7 to + 2. For several samples, there was almost no concordance across platforms. For example, in sample B09-SN21, DECODR reported 100% indel presence with a dominant − 60 base pair loss, while TIDE reported only 3% indel presence with a dominant indel of − 30 base pairs for the same sample. Synthego was unable to align this sequence, thereby providing no indel information, which is indicated by “x” . In some sequences, DECODR was discordant from both TIDE and Synthego analyses, such as in samples SN2.5 (100% cut with − 153 indel vs. 2% cut with − 26 indel) and B11-SN25 (100% cut with − 26 indel vs. 5.1% cut with − 10 indel).

To confirm the capability of DECODR to detect indels of varying sizes, we developed in silico deletions of the *Nf1* locus at 10 bp intervals from 10 to 50 bp followed by 50 bp intervals from 50 to 200 bp (Supplementary Fig. 4A). We observed that DECODR accurately detected the size of the introduced indel which further confirms the range of indel detection window for DECODR (Supplementary Fig. 4B,C).

To determine if these trends in indel complexity were observed when *p53* is targeted with our CRISPR gene-editing system, we analyzed the indels of *p53* PCR products from corresponding biological samples analyzed for *Nf1* indels. Similar to our findings with *Nf1* indels, results for *p53* indels were reported as widely divergent amounts of total indel percentage within the same sample (Supplementary Fig. 5A, left). However, the distribution of total indel percentage in each sample was comparable across the different software packages (Supplementary Fig. 5A, right). Similar trends were seen in the *p53* indel analyses as observed in *Nf1* indels with some samples demonstrating a strong concordance between indel percentage and indel size (Supplementary Fig. 5B). Taken together, this analysis shows that different software platforms can report widely divergent indel data from the same sample, particularly if larger indels are present.

Next-generation sequencing (NGS) is frequently used to measure CRISPR-generated indels, although it is cost-prohibitive for some research groups. To compare indel analysis software (TIDE, Synthego, and DECODR) capability to NGS, we sequenced our most complex *Nf1* sequences with NSG, aligned the sequences, and visualized indels. Sequencing of seven previously identified sequences showed a variety of indel patterns with some having a strong concordance to reported indels (Supplementary Fig. 6A–H). For example, SN7-5 had a predominating indel of − 2 bp that was detected in NGS, TIDE, Synthego, and DECODR (Fig. 3, Supplementary Fig. 6E). However, some samples had indels identified by NGS that were only detected via DECODR. NSG analysis of SN1-5 and SN10-4 revealed a predominating indel of − 31 bp and − 40 bp, respectively, that was not identified by the TIDE and Synthego analyses (Fig. 3, Supplementary Fig. 6A,G). Linear regression analysis showed that DECODR analysis corresponded significantly with NGS analysis compared to TIDE- or Synthego-reported indels (Supplementary Fig. 6H). Moreover, this analysis highlights the utility of DECODR for indel analysis of in vivo, somatic CRISPR models in comparison to TIDE and Synthego.

### Indel analysis software variability is different across murine background strains

Previously, we used our CRISPR/Cas9 MPNST tumorigenesis model to examine the impact of murine background strain on sarcoma growth and immune infiltration in C57BL/6, 129X, BALB/c, and 129 Sv/Jae mice. Our data showed that tumor initiation in BALB/c mice occurs earlier than in C57BL/6, 129X, and 129 Sv/Jae mice^20^. Although our initial analysis did not identify differences in indel composition across the four background strains, this prior study was limited to the TIDE software program. From our data described above, we now know that TIDE analysis can miss larger indels that are generated during in vivo tumorigenesis. Therefore, we re-evaluated the indel mutational profiles from our previously-published data to determine if genetic strain was contributing to the indel analysis software variability. To test this, *Nf1* sequence files were stratified by mouse background and reanalyzed for total indel percentage distribution and variability, in addition to number of indels, indel types, and maximum indel size identified (Supplementary Fig. 7).

**Figure 4:**
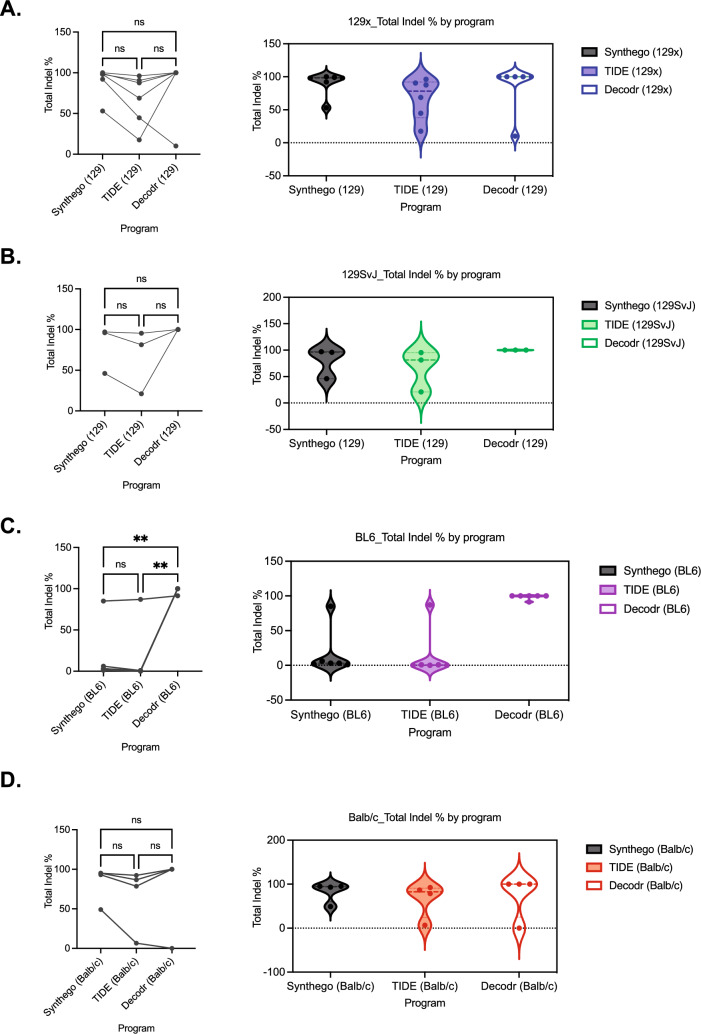
Indel analysis software variability is different across background strains. (**A**) Total indel percentage of 129 × *Nf1* sequences on Synthego, TIDE, and DECODR (n = 5). (**B**) Total indel percentage of 129SvJ *Nf1* sequences on Synthego, TIDE, and DECODR (n = 3). (**C**) Total indel percentage of C57BL/6 *Nf1* sequences on Synthego, TIDE, and DECODR (n = 6). (**D**) Total indel percentage of Balb/c *Nf1* sequences on Synthego, TIDE, and DECODR (n = 4). Variability in total indel percentage was analyzed by one-way ANOVA with Tukey’s multiple comparisons. Data represents biological replicates with the mean ± SD; *P < 0.05, **P < 0.01, ***P < 0.001, ****P < 0.0001.

Sequences from 129X and 129 Sv/Jae mice had comparable distributions between indel analysis software (Fig. 4A, left). Additionally, the total indel percentage varies widely, as indels detected by TIDE are substantially lower than Synthego (Fig. 4A, right). Sequences from C57BL/6 mice analyzed with TIDE and Synthego had comparable indel distributions, while sequences from C57BL/6 mice analyzed with DECODR clustered around 100% (Fig. 4B, left). Additionally, the total indel percentage detected by TIDE and Synthego were significantly lower than percentages identified by DECODR (Fig. 4B, right). Conversely, sequences from BALB/c mice had the least amount of variability across indel analysis software as all BALB/c sequences analyzed cluster around 100% except for one outlier sequence (Fig. 4C).

**Figure 5:**
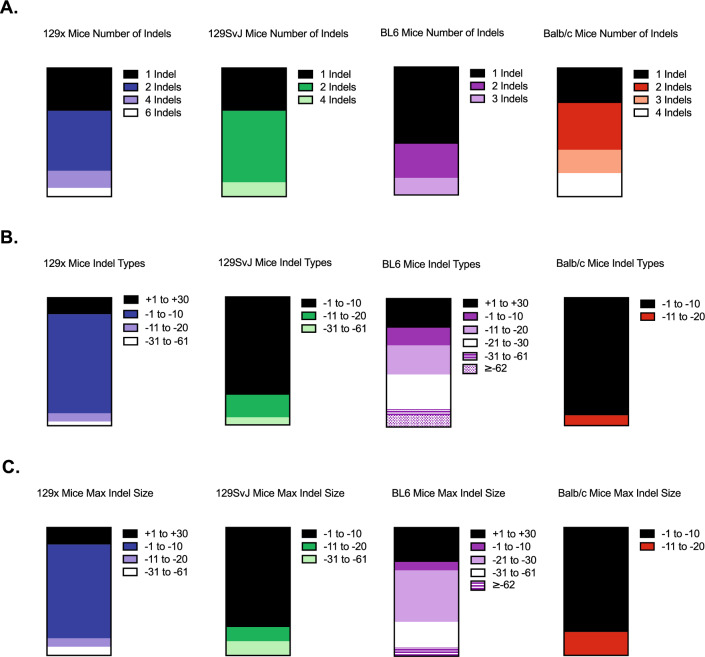
Mouse background strain correlates with detected indel size. (**A**) Global indel characterization of number of indels of *Nf1* sequences from 129x, 129SvJ, C57BL/6, and Balb/c mice (left to right). (**B**) Global indel characterization of indel type of *Nf1* sequences from 129x, 129SvJ, C57BL/6, and Balb/c mice (left to right). (**C**) Global indel characterization of maximum indel size detected of *Nf1* sequences from 129x, 129SvJ, C57BL/6, and Balb/c mice (left to right).

We next asked if this variability across inbred strains correlated with larger indel types and more complex mutational profiles. To test this, we characterized the number of indels, indel types, and maximum indel size for each mouse background strain. The number of indels detected ranged from 1 to 6 in 129X and 129 Sv/Jae mice, 1–3 in C57BL/6 mice, and 1–4 in BALB/c mice (Fig. 5A). This suggests that the variability in indel analysis software across mouse background strain is not correlated with the number of indels detected. Then, we asked if the increased variability in C57BL/6 mice total indel percentage was dependent on the indel type. The majority of indels detected in 129X and 129 Sv/Jae samples were within the 1–10 deletion range (Fig. 5B, left). Sequences from C57BL/6 mice had indel types that fell into six categories: 1–30 insertion, 1–10 deletion, 11–20 deletion, 21–31 deletion, 31–61 deletion, and 62 deletion or larger. Importantly, there was no single indel type that made up the majority of the sequences in C57BL/6 samples, suggesting an overall more complex mutational landscape in sequences from the C57BL/6 mice (Fig. 5B, middle). Sequences from BALB/c mice had a simpler indel profile that fell into two categories: 1–10 deletion and 11–20 deletion (Fig. 5B, right). The same trends observed in indel type characterizations were seen when evaluating maximum indel size (Fig. 5C).

Overall, mouse background strain did not correlate with the number of indels detected, but the size of indels did correlate with variability observed across software. Sequences from BALB/c mice had the least complex indel type composition, the smallest indels and the least amount of variability in the total indel percentage. In contrast, sequences from C57BL/6 mice had the most complex indel type composition, the largest indels and the greatest amount of variability in total indel percentage. Together these data suggest the complexity and size of indels detected are loosely correlated with total indel percent variability with C57BL/6 having the most variability in total indel percentage (Fig. 4B) and larger indel types detected (Fig. 5B). Importantly, we examined any potential genomic differences in the *Nf1* gene locus between inbred mouse strains by using Jax Laboratories inbred strain comparison resource (http://www.informatics.jax.org/home/strain). We observed that C57BL6 mice have two lncRNAs that are not present in the other inbred lines (Supplementary Fig. 8). However, these lncRNAs are not located in *Nf1* regulatory regions nor the gRNA-targeted sites (Supplementary Fig. 9A,B). Therefore, the background strain indel variability appears to involve more than DNA sequence alone. Contributors of this variability could include epigenetic mechanisms, DNA damage response, or immune involvement^6^. As CRISPR technology continues to be utilized, further research into background strain differences would provide a better understanding of these strain-dependent indel differences.

## Discussion

The clinical utility of CRISPR technology has been drastically increasing since the commercialization of CRISPR pipelines and in silico design/analysis tools. There are several active trials using CRISPR/Cas9 technology as an intervention to date. These trials are utilizing CRISPR gene editing in ex vivo settings where cells are manipulated in vitro and injected into the patient. However, somatic in vivo CRISPR gene editing is gaining popularity in developing clinically relevant mouse models, and studies are testing the utility of in vivo editing for patient interventional studies^6,29,36^. The gold standard for indel analysis in the clinical setting remains NGS^29^. As we think about screening interventions preclinically, the historical disconnect from bench to bedside, highlights a need to validate the various CRISPR tools being used in the preclinical space. In this study, we compare functional and aesthetic features across three commonly used, publicly available indel analysis software. Additionally, we provide a comprehensive comparison between indel analysis tools in our somatic CRISPR tumorigenesis mouse model. To our knowledge, this is the first comparison of its kind with implications for preclinical research efforts as well as considerations for CRISPR technology as it is harnessed for patient care.

We observed variability within *Nf1* indels, a gene characteristically mutated in neurofibromas and MPNST development, that correlated with the complexity of the indels detected as well as the mouse background strain. Similarly, *p53* indel variability appeared to correlate with the complexity of the indels. *Nf1* indel patterns either contained simple mutational landscapes containing 1 to 2 indels with indel sizes less than 20 bp or complex mutational landscapes containing 3 or more indels with indel sizes as large as 150 bp (Figs. 2, 3). Moreover, these findings were corroborated via NGS sequencing which revealed that DECODR analysis of somatic, in vivo generated indels correlated strongly to NGS reported indels compared to TIDE and Synthego (Supplementary Fig. 6). Complex mutational landscapes were observed more often in sequences from C57BL6 mice compared to the other three common inbred stains tested (Figs. 4, 5).

Based on these analyses, there are several considerations concerning resolution of indel detection when using these publicly available indel analysis software to assess the efficacy of your CRISPR/Cas9 system (Table 1). TIDE, Synthego, DECODR, and Indigo all provide sequence alignment functions. However, Indigo does not use any sequence deconvolution algorithm which makes synthesizing and interpreting indels difficult. For this reason, Indigo was not used for much of the analysis. TIDE, Synthego, and DECODR all provide alignment, sequence deconvolution, and indel contribution features. TIDE provides an adjustable alignment window with a window limit of − 50 to + 15 bp while neither Synthego nor DECODR have an adjustable alignment window. However, Synthego has a window limit of − 30 to + 14 bp while DECODR does not have a window limit. Additionally, DECODR has the capacity to input two gRNAs compared to TIDE and Synthego with only one gRNA. Previously, TIDE indel size and accuracy have been shown to correlate with Synthego indel calls with an r^2^ of 0.99. Synthego indel size and accuracy have been shown to correlate with NGS with an r^2^ of 0.93^9^. However, these indels were induced in vitro with the largest indel induced being 36 bp^9^. DECODR appears to be better equipped to provide accurate indel characterization when inducing indels in vivo and/or when indels larger than 30 bp are expected (Supplementary Fig. 6). Here, we find that reported limitations hold true for somatic, CRISPR tumorigenesis models. Furthermore, we report that the variability of indel mutational landscapes is increased in C57BL/6 mice while indels generated in 129x, 129SvJ, and Balb/c mice are more homogenous.

**Figure 6:**
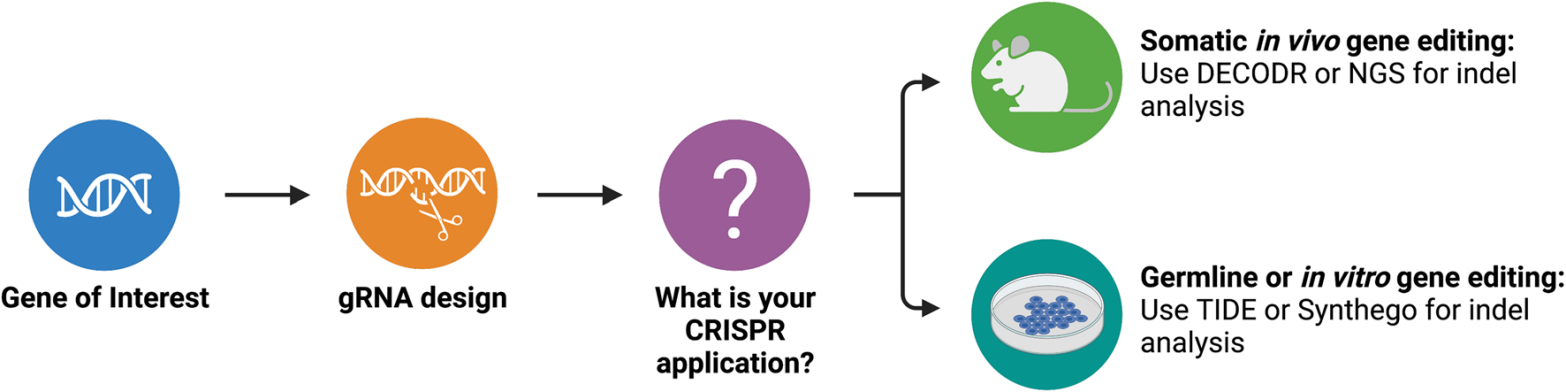
Decision tree for selecting indel analysis platforms best equipped for different applications.

Accurate detection of CRISPR/Cas9 indels induced in vivo is a question of increasing importance that requires a pre-emptive assessment of CRISPR gene-editing application and what is the best tool for your purpose. TIDE or Synthego are powerful indel analysis tools that are appropriate for germline or in vitro gene editing. DECODR is an indel analysis tool that has comparable deconvolution methods as TIDE/Synthego, but the lack of a window limit makes DECODR better equipped for somatic in vivo gene editing (Fig. 6). NGS is the method readily used in the clinic but is presently not cost-effective for most labs that use CRISPR editing at a high volume to model human disease. As of 2022, over 60 clinical trials at varying stages have a component involving a CRISPR-based intervention mainly focused on cancer therapies. Currently, the majority of these efforts are focused on ex vivo manipulation of patient cells. However, the prevalence of CRISPR technology in clinical trials highlights the need for accurate design and analysis tools as new tools are developed for clinical trials as well as therapy efficacy is screened in preclinical settings.

### Supplementary Information

Supplementary Figures.

## Data Availability

The sanger sequencing datasets generated and/or analyzed during the current study are available in the GenBank repository, BankIt2652655 OP977989-OP978006. All NGS datasets will be provided upon request.
