# Supplementary material for: Discrepancies in indel software resolution with somatic CRISPR/Cas9 tumorigenesis models
Source: Sci Rep. 2023 Sep 8;13:14798. doi: 10.1038/s41598-023-41109-1 (PMC10491828; doi:10.1038/s41598-023-41109-1)
Supplement: Supplementary file 1 — Supplementary Figures. [file 41598_2023_41109_MOESM1_ESM.pdf]

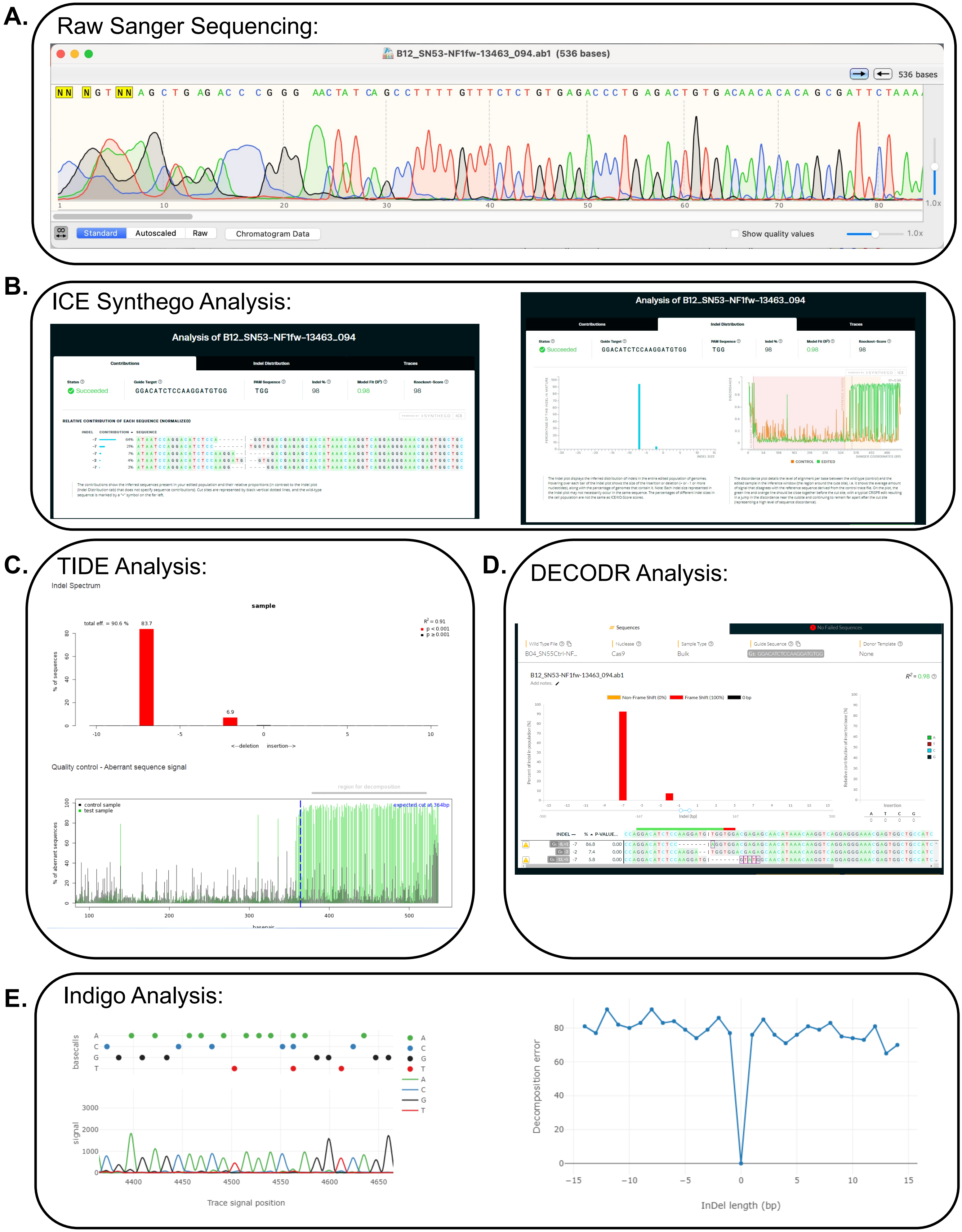
A.
Raw Sanger Sequencing:
B12_SN53-NF1fw-13463_094.ab1 (536 bases)
536 bases
Standard
Autoscaled
Raw
Chromatogram Data
Show quality values
B.
ICE Synthego Analysis:
Analysis of B12_SN53-NF1fw-13463_094
Contributions
Indel Distribution
Traces
Succeeded
GGACATCTCCAAGGATGTGG
TGG
98
0.98
98
C.
TIDE Analysis:
Indel Spectrum
sample
total eff. = 90.6 %
83.7
6.9
R^2 = 0.91
p < 0.001
p ≥ 0.001
% of sequences
<--deletion insertion-->
Quality control - Aberrant sequence signal
region for decomposition
control sample
test sample
expected cut at 364bp
% of aberrant sequences
basepair
D.
DECODR Analysis:
Sequences
No Failed Sequences
B04_SN55Ctrl-NF...
Cas9
Bulk
GGACATCTCCAAGGATGTGG
None
B12_SN53-NF1fw-13463_094.ab1
R^2 = 0.98
Non-Frame Shift (0%)
Frame Shift (100%)
0 bp
Indel (bp)
Insertion
E.
Indigo Analysis:
basecalls
signal
Trace signal position
Decomposition error
InDel length (bp)

Supplemental Figure 1: Indel analysis software have various outputs. (A) Representative raw sanger sequence following cell line generation, genomic DNA harvest, Nf1 amplification, and sequencing. (B) Representative analysis output from ICE. (C) Representative analysis output from TIDE. (D) Representative analysis output from DECODR. (E) Representative analysis output from Indigo.

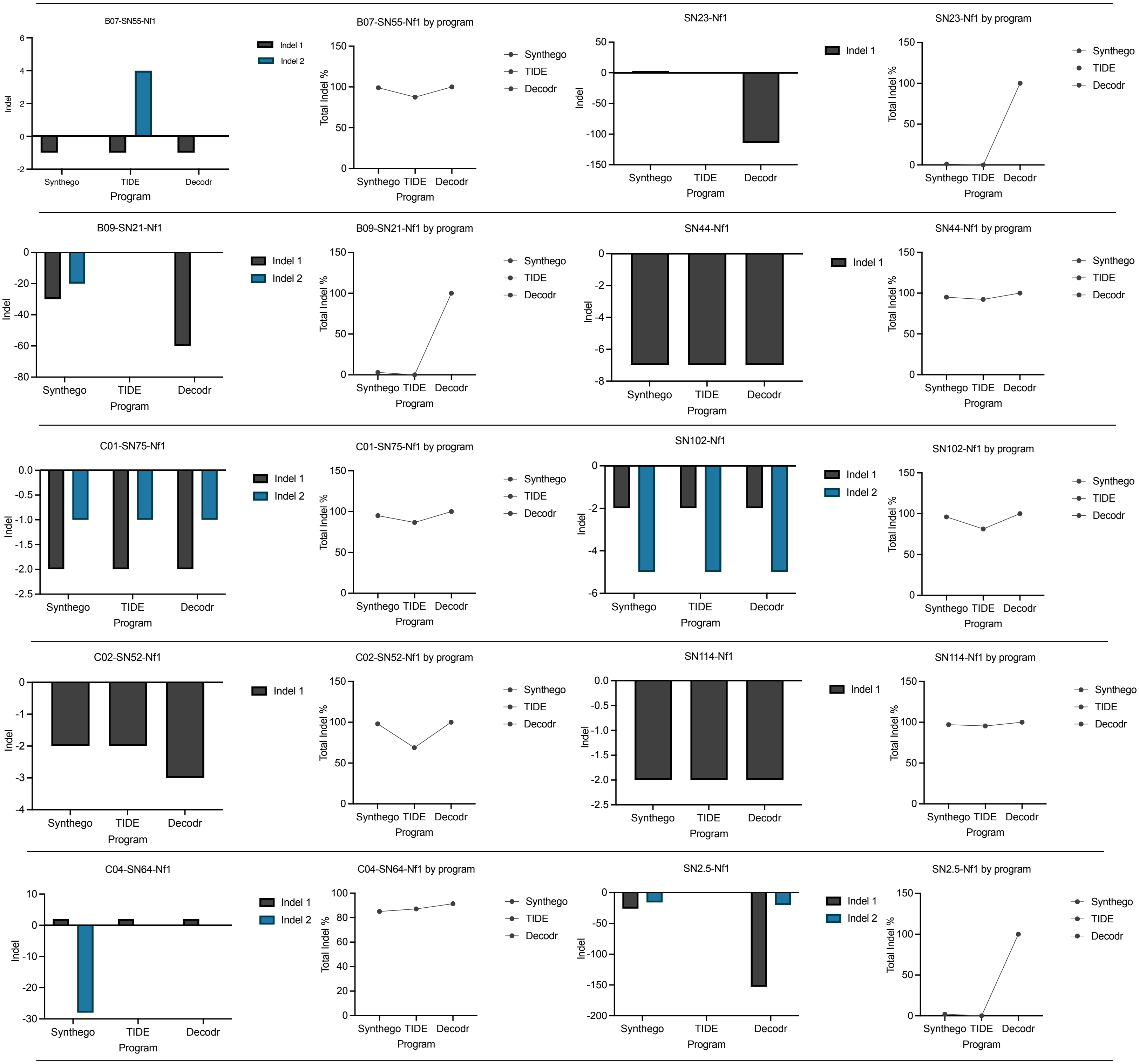

Supplemental Figure 2: Mutational landscapes with 1-2 indels have less variability between indel analysis software. Indel characterization of a Nf1 sequence with a simple mutational landscape of 1-2 indels detected (n=10).

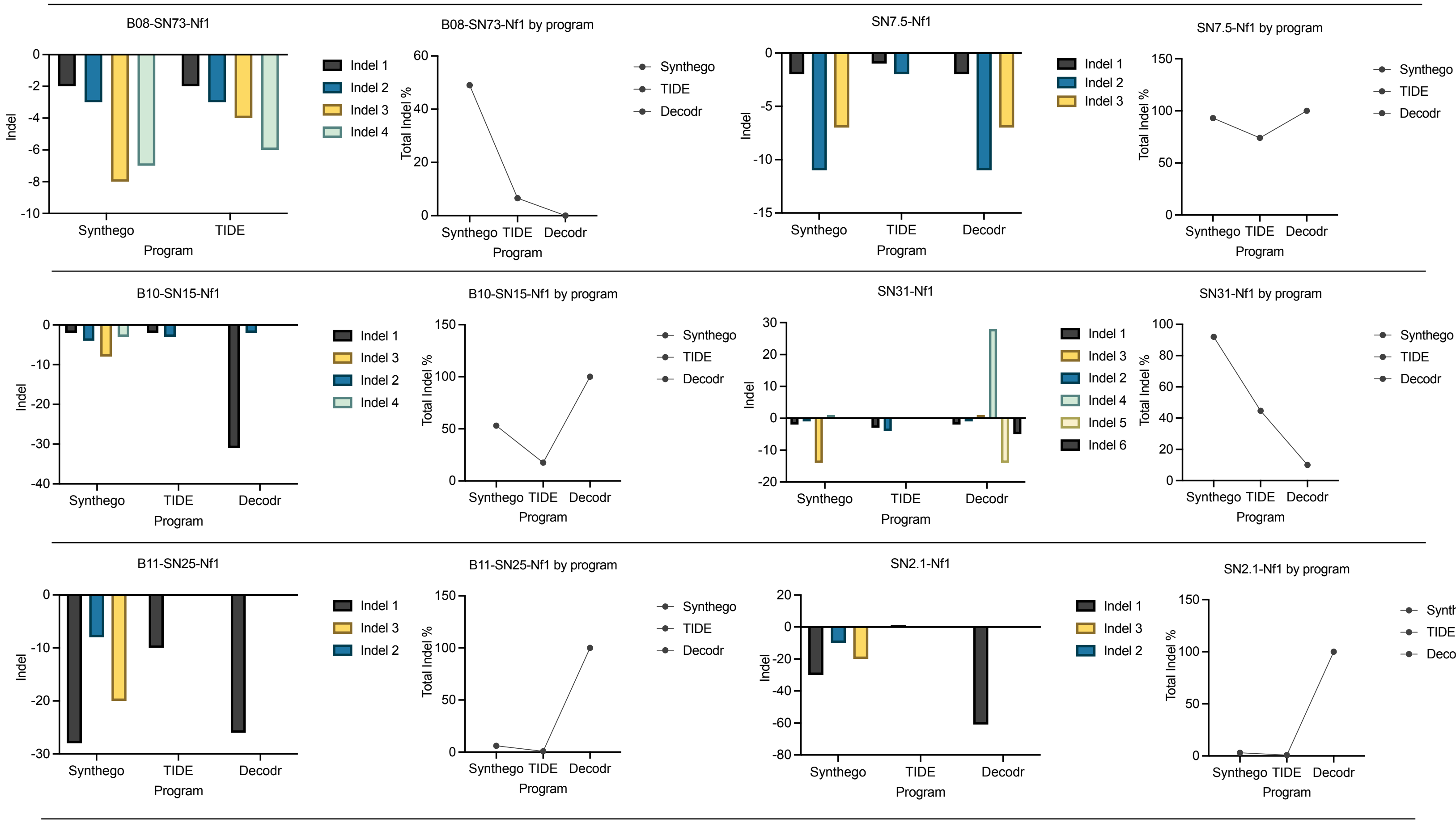

Supplemental Figure 3: Mutational landscapes with ≥3 indels have more variability between indel analysis software. Indel characterization of a Nf1 sequence with a complex mutational landscape of ≥3 indels detected (n=6).

A.

Neurofibromin 1 (Nf1): ENSMUSG00000020716 Protein coding

PROGRAM	INDELS	INDEL%	INDEL TYPE	R-SQUARED	TOTAL INDEL %
DECODR	-10	100%	Frame-shift	1.0	100%
	-20	100%	Frame-shift	1.0	100%
	-30	100%	non-shift	1.0	100%
	-40	100%	Frame-shift	1.0	100%
	-50	100%	Frame-shift	1.0	100%
	-100	100%	Frame-shift	1.0	100%
	-150	100%	Frame-shift	1.0	100%
	-200	100%	Frame-shift	1.0	100%

B.

DECODR Analysis:

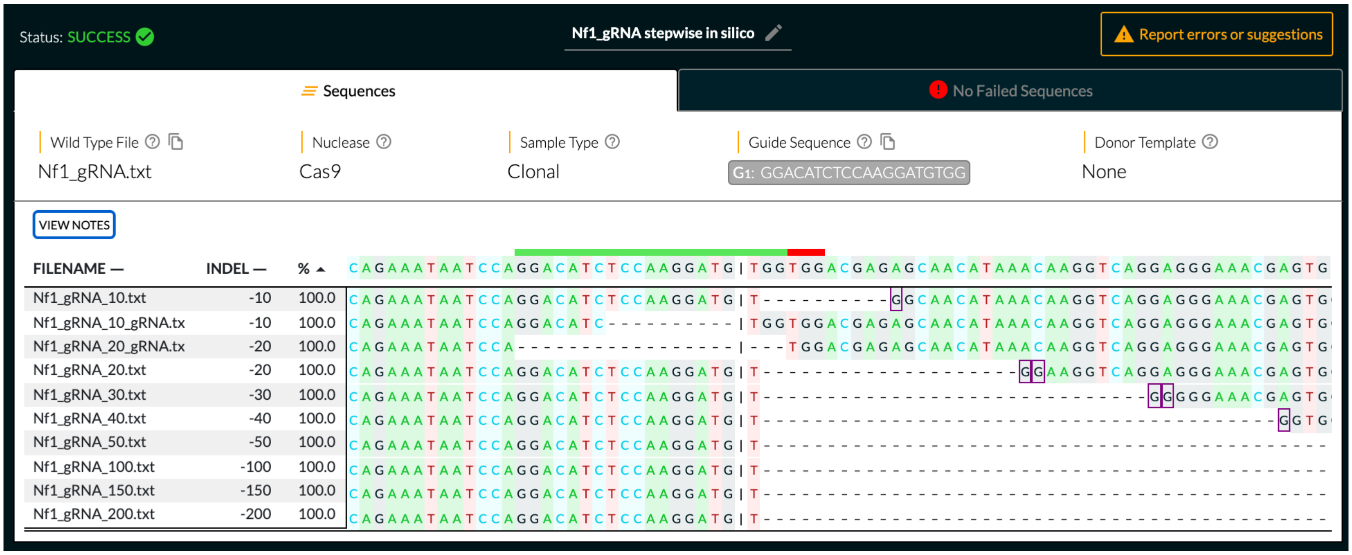

C.

DECODR Analysis: -10bp

DECODR Analysis: -20bp

DECODR Analysis: -30bp

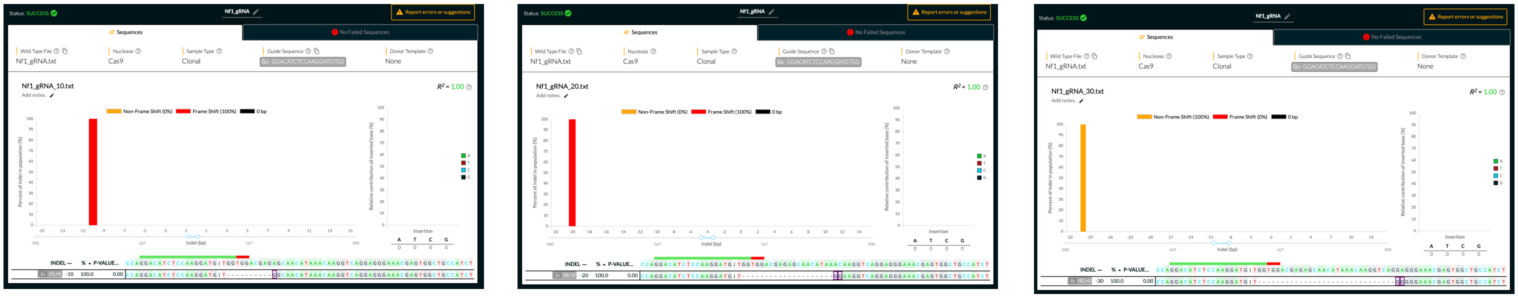

Supplemental Figures 4: *In silico* DECODR Nf1 controls. The Nf1 *Mus musculus* sequence was downloaded in .txt format and saved with *in silico* generated indels (-10, -20, -30, -40, -50, -100, -150, and -200bp). (A) DECODR output of the entire group of Nf1 sequences. (B) Individual analysis output for -10, -20, and -30bp indels.

A.

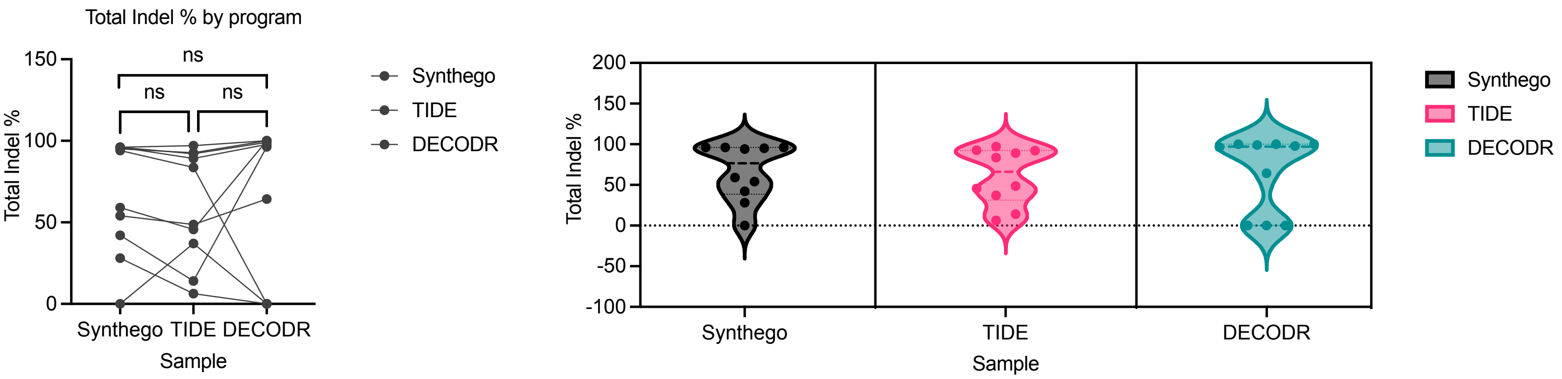

B.

Indel Summary Indel %			
Sample ID	TIDE	Synthego	DECODR
A05-SN55-p53fw	37.1	x	x
A06-SN73-p53fw	6.3	28	x
A07-SN21-p53fw	83.7	94	x
A08-SN15-p53fw	14	42	96.3
A09-SN25-p53fw	89.2	96	97.8
A10-SN53-p53fw	92.1	96	99
A11-SN75-p53fw	92.7	95	100
A12-SN35-p53fw	97	96	100
B01-SN52-p53fw	45.7	59	100
B02-SN64-p53fw	48.7	54	64.3

Indel Summary Indel Size			
Sample ID	TIDE	Synthego	DECODR
A05-SN55-p53fw	-2	x	x
A06-SN73-p53fw	-3	-20	x
A07-SN21-p53fw	-9	-9	x
A08-SN15-p53fw	-1	-38	-50
A09-SN25-p53fw	-1	-1	-1
A10-SN53-p53fw	-7	-7	-7
A11-SN75-p53fw	-8	-8	-8
A12-SN35-p53fw	-6	-6	-6
B01-SN52-p53fw	-10	-32	-263
B02-SN64-p53fw	-8	-27	-27

Green=same; Blue=within 10; Purple=11-25; Red 26+

Supplemental Figure 5: Mutational landscape of p53 correlates with the variability of indel analysis software. (A) Total indel percentage of *Nf1* sequences on Synthego, TIDE, and DECODR (n=10). Variability in total indel percentage was analyzed by one-way ANOVA with Tukey's multiple comparisons. (B) Total indel percentage and indel size for each sequence for all three platforms. Total indel percentages and indel sizes are color coded with different circle colors: green circles indicate the value was the same across platforms, blue circles indicate the value was within 10 across platforms, purple circles indicate the value was between 11 and 25 from other platforms, and red circles indicate a variation greater than 26.

A.

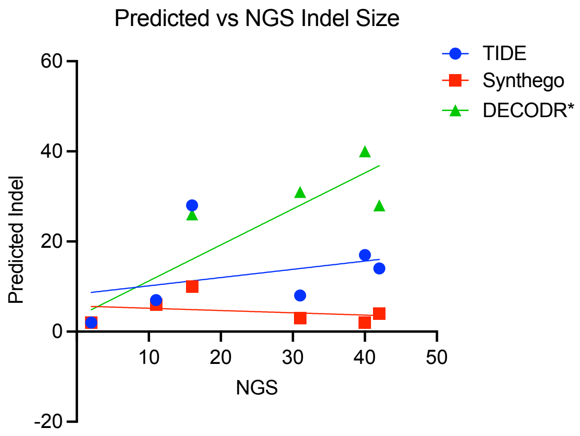

Predicted vs NGS Indel Size
Predicted Indel
NGS
60
40
20
0
-20
10
20
30
40
50
TIDE
Synthego
DECODR*

B.

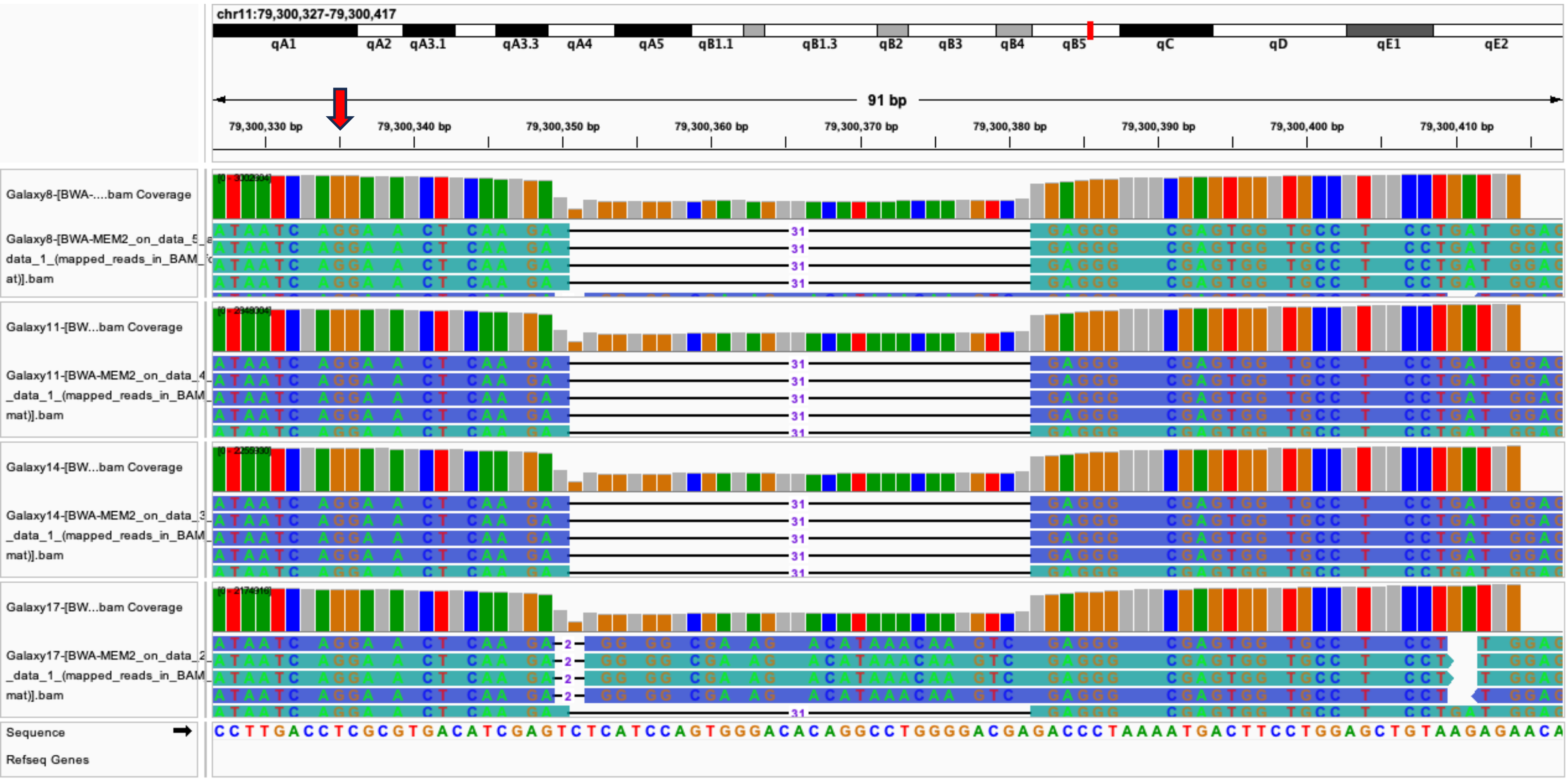

chr11:79,300,327-79,300,417
qA1
qA2
qA3.1
qA3.3
qA4
qA5
qB1.1
qB1.3
qB2
qB3
qB4
qB5
qC
qD
qE1
qE2
91 bp
79,300,330 bp
79,300,340 bp
79,300,350 bp
79,300,360 bp
79,300,370 bp
79,300,380 bp
79,300,390 bp
79,300,400 bp
79,300,410 bp
Galaxy8-[BWA-....bam Coverage
Galaxy11-[BW...bam Coverage
Galaxy14-[BW...bam Coverage
Galaxy17-[BW...bam Coverage
Sequence
Refseq Genes
CCTTGACCTCGCGTGACATCGAGTCTCATCCAGTGGGACACAGGCCTGGGGACGAGACCCTAAAATGACTTCCTGGAGCTGTAAGAGAACA

C.

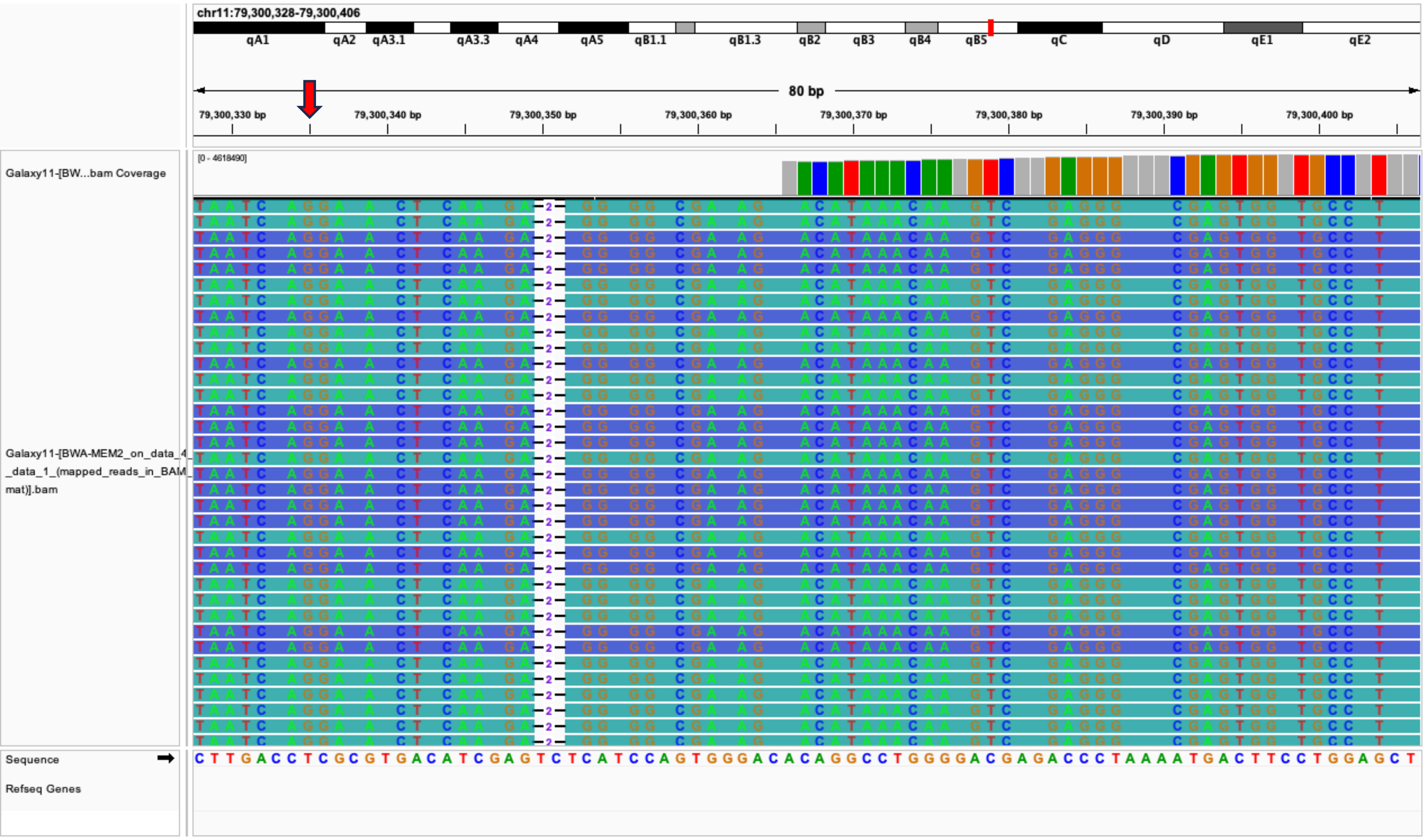
chr11:79,300,328-79,300,406
qA1 qA2 qA3.1 qA3.3 qA4 qA5 qB1.1 qB1.3 qB2 qB3 qB4 qB5 qC qD qE1 qE2
80 bp
79,300,330 bp
79,300,340 bp
79,300,350 bp
79,300,360 bp
79,300,370 bp
79,300,380 bp
79,300,390 bp
79,300,400 bp
Galaxy11-[BW...bam Coverage
[0 - 4618490]
Galaxy11-[BWA-MEM2_on_data_4_
_data_1_(mapped_reads_in_BAM_
mat)].bam
Sequence
Refseq Genes

D.

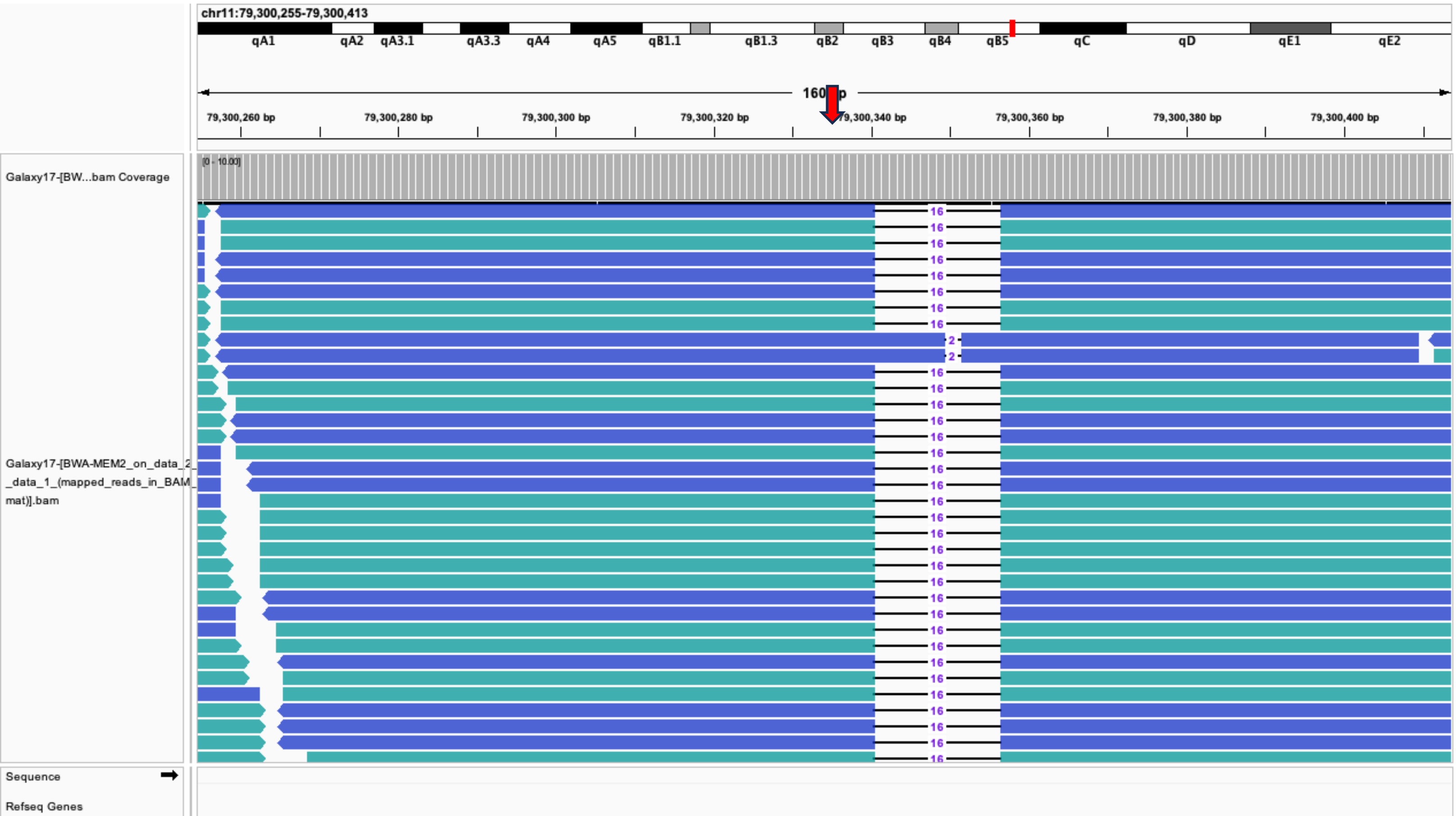
chr11:79,300,255-79,300,413
qA1 qA2 qA3.1 qA3.3 qA4 qA5 qB1.1 qB1.3 qB2 qB3 qB4 qB5 qC qD qE1 qE2
79,300,260 bp
79,300,280 bp
79,300,300 bp
79,300,320 bp
79,300,340 bp
79,300,360 bp
79,300,380 bp
79,300,400 bp
Galaxy17-[BW...bam Coverage
[0 - 10.00]
Galaxy17-[BWA-MEM2_on_data_2_
_data_1_(mapped_reads_in_BAM_
mat)].bam
Sequence
Refseq Genes

E.

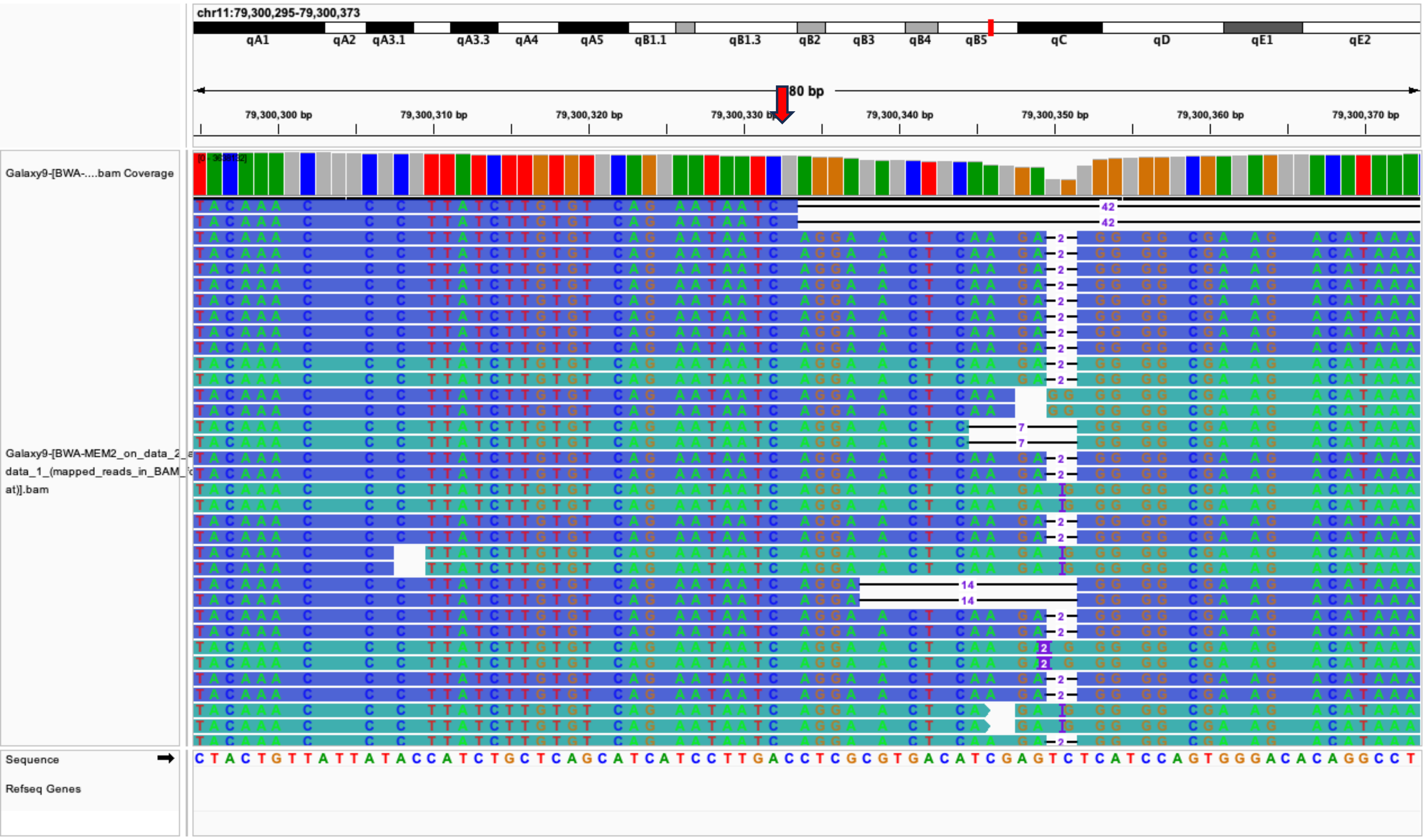
chr11:79,300,295-79,300,373
qA1 qA2 qA3.1 qA3.3 qA4 qA5 qB1.1 qB1.3 qB2 qB3 qB4 qB5 qC qD qE1 qE2
80 bp
79,300,300 bp
79,300,310 bp
79,300,320 bp
79,300,330 bp
79,300,340 bp
79,300,350 bp
79,300,360 bp
79,300,370 bp
Galaxy9-[BWA-....bam Coverage
Galaxy9-[BWA-MEM2_on_data_2_a
data_1_(mapped_reads_in_BAM_fo
at)].bam
42
2
7
14
Sequence
Refseq Genes
CTACTGTTATTATACCATCTGCTCAGCATCATCCTTGACCTCGCGTGACATCGAGTCTCATCCAGTGGGACACAGGCCT

F.

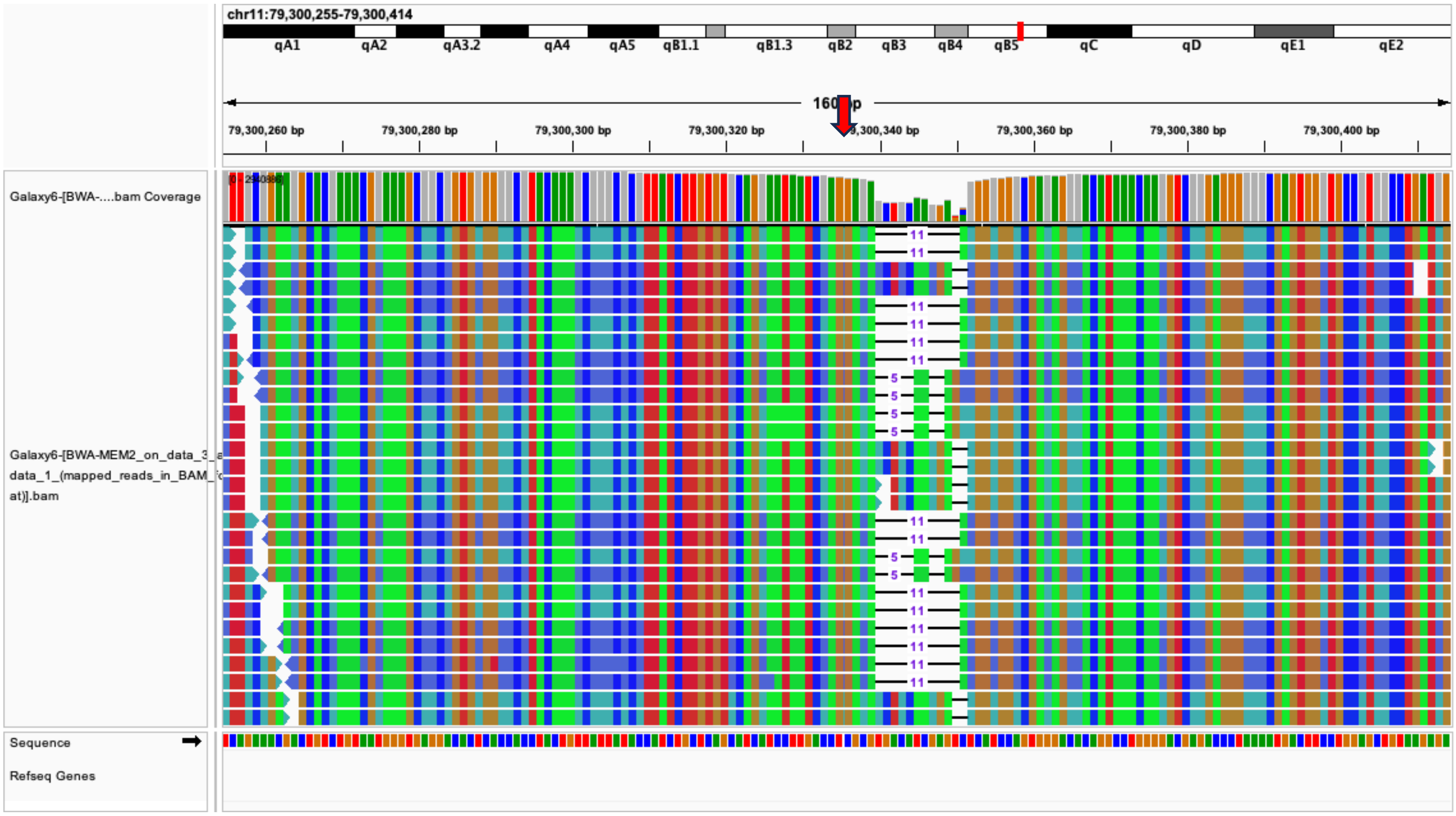
chr11:79,300,255-79,300,414
qA1 qA2 qA3.2 qA4 qA5 qB1.1 qB1.3 qB2 qB3 qB4 qB5 qC qD qE1 qE2
160 bp
79,300,260 bp
79,300,280 bp
79,300,300 bp
79,300,320 bp
79,300,340 bp
79,300,360 bp
79,300,380 bp
79,300,400 bp
Galaxy6-[BWA-....bam Coverage
Galaxy6-[BWA-MEM2_on_data_3_a
data_1_(mapped_reads_in_BAM_fo
at)].bam
11
5
Sequence
Refseq Genes

G.

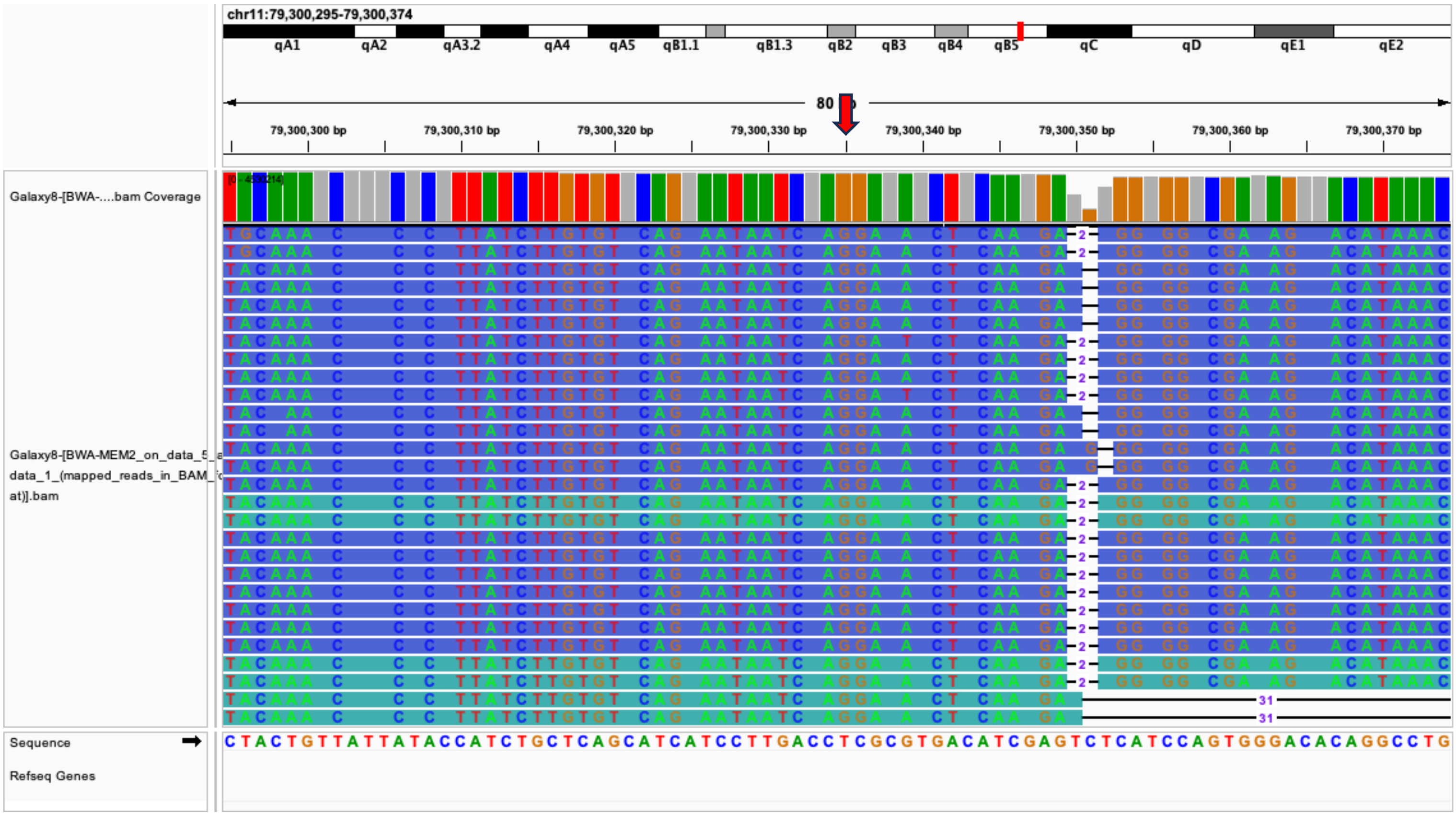

chr11:79,300,295-79,300,374
qA1 qA2 qA3.2 qA4 qA5 qB1.1 qB1.3 qB2 qB3 qB4 qB5 qC qD qE1 qE2
80 bp
79,300,300 bp
79,300,310 bp
79,300,320 bp
79,300,330 bp
79,300,340 bp
79,300,350 bp
79,300,360 bp
79,300,370 bp
Galaxy8-[BWA-....bam Coverage
Galaxy8-[BWA-MEM2_on_data_5_a data_1_(mapped_reads_in_BAM_f at)].bam
Sequence
Refseq Genes
CTACTGTTATTATACCATCTGCTCAGCATCATCCTTGACCTCGCGTGACATCGAGTCTCATCCAGTGGGACACAGGCCTG

H.

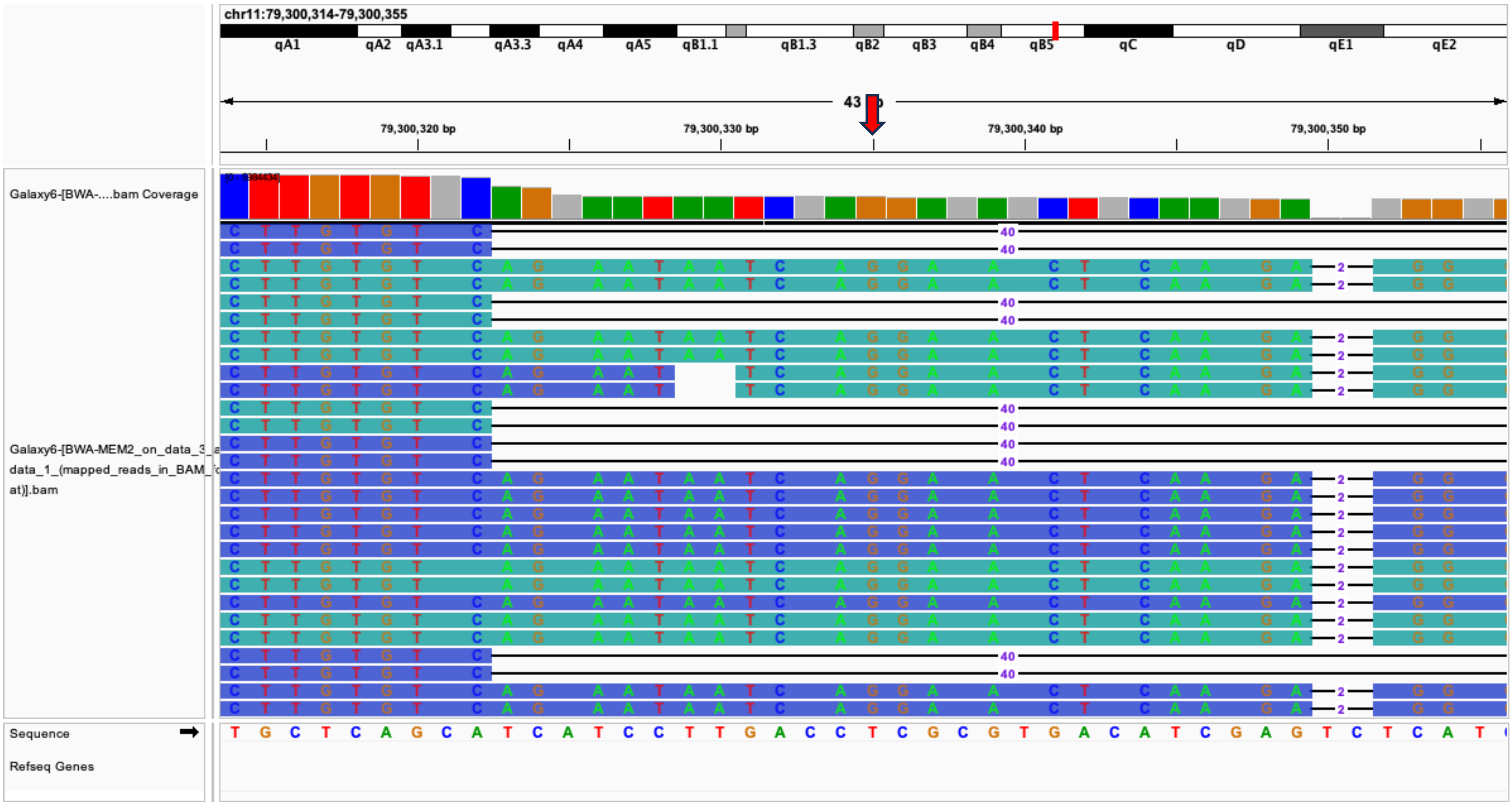

chr11:79,300,314-79,300,355
qA1 qA2 qA3.1 qA3.3 qA4 qA5 qB1.1 qB1.3 qB2 qB3 qB4 qB5 qC qD qE1 qE2
43 bp
79,300,320 bp
79,300,330 bp
79,300,340 bp
79,300,350 bp
Galaxy6-[BWA-....bam Coverage
Galaxy6-[BWA-MEM2_on_data_3_a data_1_(mapped_reads_in_BAM_f at)].bam
Sequence
Refseq Genes

Supplemental Figure 6: Next generation sequencing (NGS) of mutational landscapes with ≥3 indels identified via indel analysis software. (A) Linear regression of absolute indel size identified via NGS compared to TIDE, Synthego, and DECODR. (B-H) IGV alignments of sequences with identified complex mutational landscapes (SN1-5 (B), SN2-1 (C), SN2-5 (D), SN3-1 (E), SN7-3 (F), SN7-5 (G), and SN10-4 (H)). CRISPR gRNA sequence location is indicated with a red arrow pointed at the first 5' base pair (bp) in the sequence. Data represents biological replicates; $^*P < 0.05$.

Mouse Background Strains

Sample ID	Background
B07-SN55-Nf1fw	129x
B08-SN73-Nf1fw	Balb
B09-SN21-Nf1fw	BL6
B10-SN15-Nf1fw	129x
B11-SN25-Nf1fw	BL6
B12-SN53-Nf1fw	129x
C01-SN75-Nf1fw	Balb
C02-SN35-Nf1fw	129x
C03-SN52-Nf1fw	129x
C04-SN64-Nf1fw	BL6
SN23-Nf1fw	BL6
SN31Nf1-fw-P1	129x
SN44-Nf1fw-P1	Balb
SN102NF1-Nf1fw-P1	129SvJ
SN104NF1-Nf1fw-P1	129SvJ
SN114NF1-Nf1fw-P1	129SvJ
SN2.1NF1-NF1fwd	BL6
SN2.5NF1-NF1fwd	BL6
SN7.5NF1-NF1fwd	Balb

Supplemental Figure 7: Mouse background strain breakdown. Mouse background strain data for Nf1 sequences analyzed.

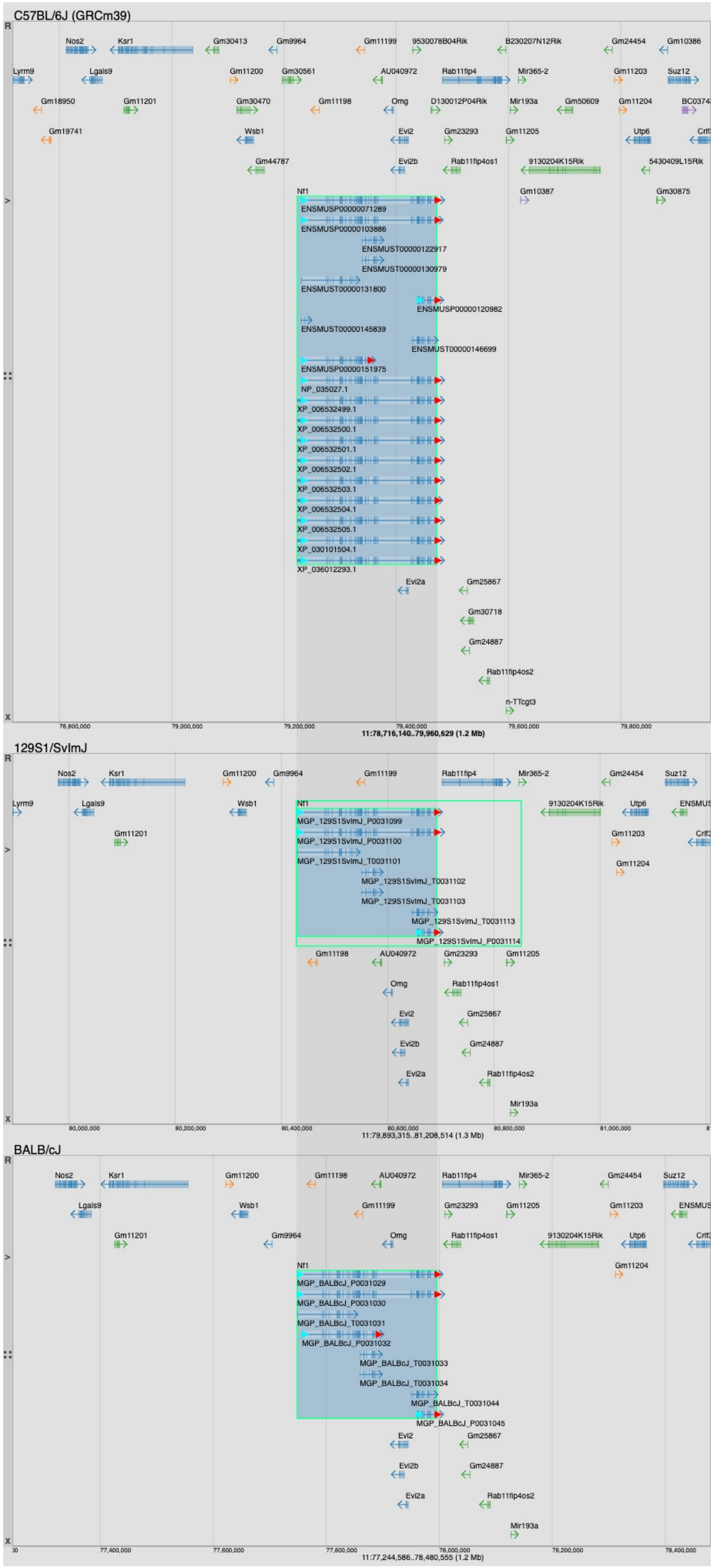

Supplemental Figure 8: Nf1 gene across mouse background strains. Nf1 gene sequence map in 129SvJ, C57BL/6, and Balb/c mice.

A.

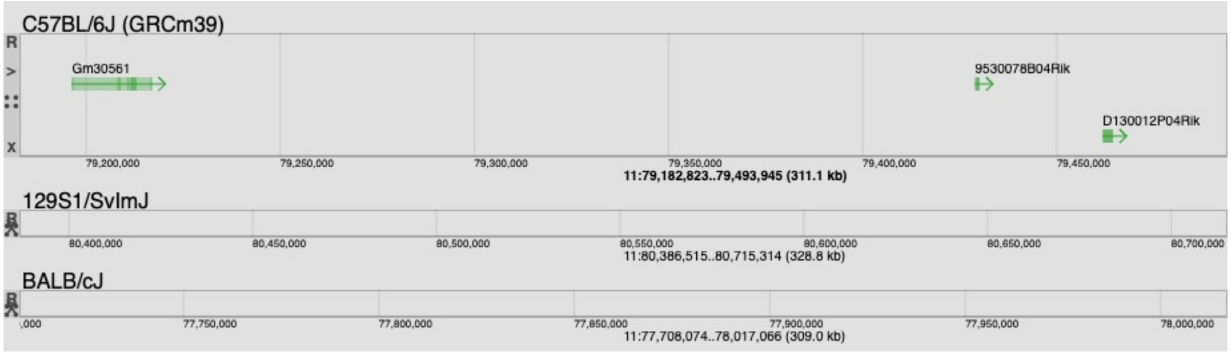

B.

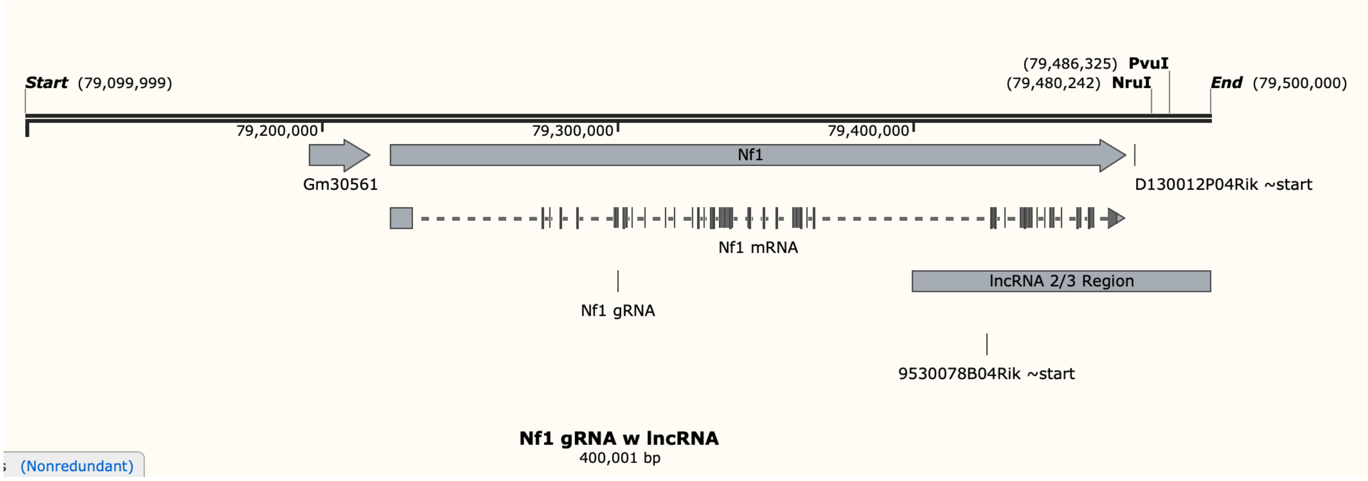

Supplemental Figure 9: C57BL/6 specific lncRNA associated with Nf1 gene. (A) Identification of three C57BL/6 specific lncRNA associated with Nf1 gene sequence. (B) Identification of three C57BL/6 specific lncRNA associated with Nf1 gene sequence in relation to Nf1 gRNA targeted sequence in CRISPR/Cas9 tumorigenesis model.
